# The Important Role of Carbohydrates in the Flavor, Function, and Formulation of Oral Nutritional Supplements

**DOI:** 10.3390/nu10060742

**Published:** 2018-06-08

**Authors:** Smaro Kokkinidou, Devin Peterson, Tama Bloch, Ashley Bronston

**Affiliations:** 1 FONA International, Geneva, IL 60134, USA; skokkinidou@fona.com; 2 Flavor Research and Education Center, The Ohio State University, Columbus, OH 43210, USA; peterson.892@osu.edu; 3 Abbott Nutrition, Columbus, OH 43219, USA; tama.bloch@abbott.com; 4 Nutrition Consultant, Abbott Nutrition, Columbus, OH 43219, USA

**Keywords:** oral nutritional supplements, carbohydrates, added sugar, nutrition policy, nutrition labeling

## Abstract

Patients who are malnourished or at-risk for malnutrition often benefit from the consumption of oral nutritional supplements (ONS). ONS supply a range of micro- and macro-nutrients, and they can be used to supplement a diet or provide total nutrition. Since ONS are specially formulated products, all ONS ingredients—including carbohydrates—are added ingredients. This may seem to be at odds with the growing public health discourse on the need to reduce “added sugars” in the diet. However, carbohydrate is an essential nutrient for human health and is a critical ingredient in ONS. Helping to educate patients on the value of “added sugars” in ONS may be useful to improve compliance with nutritional recommendations when ONS are indicated. This perspective paper reviews the important roles of “added sugars” in ONS, in terms of flavor, function, and product formulation.

## 1. Introduction

Many patients who are malnourished or at-risk for malnutrition can be managed with a variety of dietary approaches, including texture modification, fortification, increased frequency of meals/snacks, and/or the use of commercially available products, known within healthcare as oral nutritional supplements (ONS) [1]. ONS products are increasingly being recognized as an integral part of the overall patient management strategy for malnutrition, both in healthcare institutions and in the community [2]. Scientific evidence demonstrates that use of ONS can lead to improvements in nutritional intake, in addition to improvements in clinical, economic, and other outcomes [2].

ONS are designed for patients who are unable to meet their nutritional requirements through an oral diet alone [3]. These products are available for clinical use as ready-made, nutrient-dense liquids or powders [3]. In the ONS product category, some general products are used to help meet the nutritional needs of patients with various medical conditions (e.g., malnutrition and frailty), whereas other ONS are designed for very specific conditions (e.g., critical care) or disease states (e.g., diabetes and renal disease) [3]. Not all ONS products are appropriate for every patient. The interdisciplinary medical team, including dietitians, must work together to determine the correct product based on the individualized needs of the patient. In this paper, we focus on the general type of ONS that are used by patients who need extra calories, with a balanced combination of protein, fat, and carbohydrate, to improve their at-risk or malnourished condition as quickly as possible. This represents the majority of ONS patient users; generally, these patients do not have confounding metabolic conditions that can impact dietary carbohydrate recommendations or utilization.

ONS are formulated nutrition products, and therefore, all the ingredients in ONS—including the carbohydrate—are “added”. The carbohydrate sources used in ONS vary, but they are often relatively simple sugars that are easy to digest and absorb [4]. Because the carbohydrate sources are simple sugars that are “added,” public health concerns about limiting “added sugars” in the diet may bring questions from some patients. Helping to educate patients on the value of added carbohydrate or sugar in ONS may be useful to improve compliance with nutrition recommendations when ONS are indicated. This paper provides information that may be helpful for healthcare and patient education.

## 2. Health Policy Framework for Added Sugars

The health policy framework for added sugars has been driven largely by the global obesity epidemic and the rise in diet-related diseases [5]. There has been growing public health discussion on the topic of sugar consumption, particularly the overconsumption of sugars added to foods and beverages [6,7]. Public health advocates have called on consumers to limit added sugar consumption and for manufacturers to limit added sugar as an ingredient in their food products [8].

In 2010, the Dietary Guidelines for Americans (DGA) included a recommendation to reduce intake of calories from added sugars [9]. In 2015, the World Health Organization (WHO) and the 2015–2020 DGA took this a step further by recommending consumption of less than 10% of total energy intake from added sugars [10,11]. The WHO issued a further conditional recommendation to reduce consumption of free sugar to less than 5% of total energy [12].

**Figure 1:**
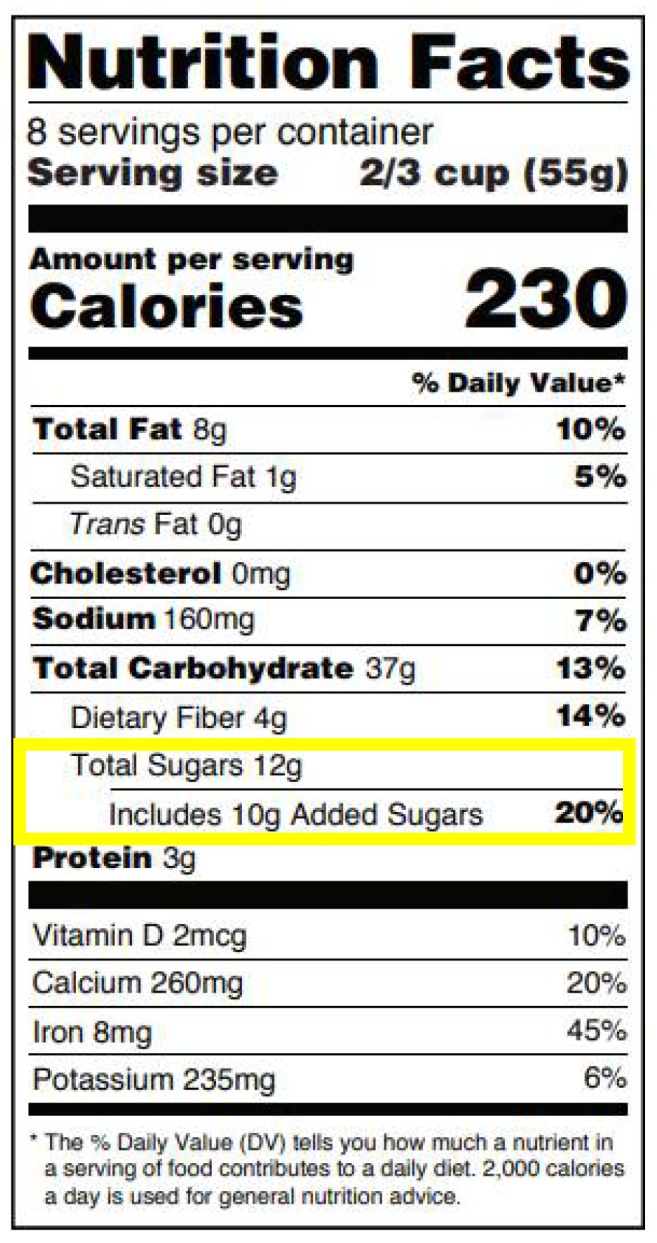
The new Nutrition Facts label with line designated for added sugar indented directly below “Total Sugars” [13].

Nutrition labelling has emerged as a policy tool in the U.S. to promote nutrition guidelines. On May 26, 2016, when the U.S. Food and Drug Administration (FDA) announced its updated design for the Nutrition Facts label for packaged foods [13], it addressed the DGA added sugar recommendation by designating a new line on the label for added sugars that would appear indented directly below “Total Sugars”, a line previously just labeled as “Sugars” [13] (See Figure 1). The purpose of this specific labeling change was to improve ingredient transparency for the consumer [5].

## 3. Added Carbohydrate in ONS

General ONS are categorized as food and thus carry a Nutrition Facts label. However, unlike traditional foods, ONS are developed and manufactured for patients who are unable to meet their nutritional requirements through a regular diet alone [3]. Furthermore, while ONS products are frequently used to supplement a diet, they can also be used to provide an individual’s total nutritional needs [14,15] or to bridge periods of nutritional inadequacy until normal oral intake of regular food can resume.

As explained in more detail in this paper, patients need a source of carbohydrate in their diet, and in ONS, the carbohydrate is essentially all added carbohydrate, as these are formulated products. The U.S. Dietary Reference Intakes (DRI) recommend that individuals consume between 45% to 65% of calories from carbohydrate, 10% to 35% from protein, and 20% to 35% from fat [16], which is similar to the proportions of macronutrients that ONS generally provide [15]. For patients with compromised oral intake, the energy and nutrient density of ONS can be highly advantageous [17]. Significantly lowering the carbohydrate content in ONS to meet the general recommendation of 10% of calories from added sugars would no longer meet the DRI’s recommended level of carbohydrate in the diet, and it would necessitate increasing the amount of protein and/or fat in the ONS beyond recommended levels to provide a product with equivalent calories. Along with concerns about no longer meeting recommended macronutrient levels, there is some evidence that ONS with higher fat content may decrease food consumption, negatively impacting overall energy intake [18].

Furthermore, the added carbohydrate in ONS has additional indispensable roles in terms of flavor, function, and formulation, which cannot be replicated by other nutrients or constituents. The anabolic effects of simple sugars, which may be disadvantageous for the general population, may be beneficial for malnourished patients and may outweigh other potential impacts, such as potential inflammatory effects. Novel carbohydrate-containing ingredients are being studied, but it is not within the scope of this paper to discuss this developing technology. In addition, manufacturers have used naturally occurring, non-nutritive sweeteners to lower the added sugars content of some ONS, but added sugars remain fundamental as core nutritional and functional product ingredients, as detailed below.

## 4. Taste and Flavor Roles of Carbohydrate in ONS

For ONS, taste and flavor are key influencing factors for compliance [19]. For ONS to provide maximum benefits, patients need to be compliant with healthcare professional recommendations on product usage. Estimates of non-compliance with ONS can vary depending on the setting. A systematic review [20], which pooled the results of 46 studies involving 4328 patients, found a mean compliance rate of 78% (range 37–100%). An ONS product is only as effective as the patient’s desire to consume it, and typically, few patients will sacrifice taste and consume an ONS product for its nutritional value alone [21].

As a species, our sense of taste has developed as a protective mechanism [22] with the basic taste attributes (sweet, salty, bitter, sour, and savory or umami) facilitating recognition and ensuring intake of safe and nutritious foods [23]. A bitter or sour taste is believed to be an indication of poisonous, inedible plants or of rotting, protein-rich food, and sweet and salty tastes were often a sign of safe, nutrient-rich foods [23]. The innate attraction to sweet taste, and in turn, the consumption of energy-rich sugars and carbohydrates, is thought to be the evolutionary trait that fueled the advancement of our species and supported the metabolic demands of our large brains [24]. The taste umami indicated a good source of protein, as it naturally occurs in animal foods, and salty indicated a good source of vital minerals [25]. Evolutionary pressures essentially drove the development of our taste receptors such that we prefer sweet and salty foods. The learned consequences of ingested foods have subsequently guided our food choices.

Taste perception is stimulated when nutrients or other chemical compounds bind to specialized receptor cells within the oral cavity [19]. Taste serves two main functions: it enables the evaluation of foods for toxicity and nutrients, and it prepares the metabolic functions of the body after ingestion. Taste perception will drive a primal sense of which sampled foods are “acceptable” or “unacceptable” [22]. Taste (gustation), aroma (olfaction), and somatosensory stimuli all contribute to the multi-model sensation of flavor. These sensations are then relayed to the brain where they are processed with other information, such as temperature, appearance (e.g., color/glossiness), shape, and sound. This sensory experience is further influenced by environment, culture, and mood, among other elements [26].

The perception of taste changes during the lifespan. For newborns, the taste sense is the most important and most developed of all senses [25]. Babies have an innate preference for sweet tastes, as this conditions them to enjoy breast milk, which contains 40% of its energy as lactose [19,27]. As people age, the taste buds begin to degenerate, and loss of taste becomes apparent [28]. Cooper et al. [29] found that taste perception remains somewhat unaffected until the late fifties, but in later years a sharp reduction is observed.

For the older adult population, taste thresholds for sweetness, saltiness, and bitterness have been reported to be at least 2.5 times higher (less sensitive) compared to younger consumer groups [30,31,32]. This altered perception can lead to significantly different food choices and reduced nutritional intake. Taste is also an important part of the cephalic phase response that prepares the body for digestion. It helps modulate food choice and meal size by increasing satiety and the pleasure of eating [33]. Loss of taste is common in the elderly and can be exacerbated by disease and drugs [33,34]. Consequently, a formidable health threat caused by the decline of taste perception in older adults is food anhedonia, or the inability to experience enjoyment, which results in reduced food consumption and ultimately the loss of body mass [35]. Studies show how important taste alterations can be; for example, compared to a young cohort, the average detection thresholds for elderly individuals having one or more medical conditions and taking three medications were 2.7 times higher for sweetness and 11.6 times higher for sodium salts [32,33,36]. The progressive consequence of taste alterations can potentially lead to malnutrition and other serious health consequences.

As mentioned above, a decline in taste perception can additionally be influenced by an increase in drug consumption with age. In the U.S., the mean number of medications used by older adults (those over the age of 65) ranges from 2.9 to 3.7 [37]. Over 250 commonly used drugs (consumed by elderly populations) have been reported to clinically affect the sensation of taste [38,39]. In addition to physiological changes associated with the aging process and drug use, taste dysfunction can be caused by additional pathological conditions such as, oral diseases [40], cancer, or systemic diseases of the central nervous system, endocrine system, cardiovascular system, or renal system [41,42,43]. A well-designed ONS with a moderately strong sweetness profile can help offset changes in taste perception that may occur due to aging, medication use, and/or disease states. A few studies have been conducted that show that improving the flavor of the foods can improve nutritional intake and increase body weight in hospital and nursing home patients as well as in the healthy elderly [44,45]. Increasing positive hedonics such as sweetness could provide an improved taste profile and increase the pleasure of eating.

## 5. Functional Roles of Carbohydrate in ONS

No single naturally-occurring food exists that delivers complete nutrition and can be administered orally, in small volumes, to nutritionally vulnerable populations. Decades of clinical research and development have optimized ONS formulations to fill this void. Carbohydrate, protein, and fat must inherently be part of the mix, each with important functional roles. In terms of carbohydrate’s functional roles in these products, foremost is its role in providing energy [46]. Carbohydrate in the diet, such as from ONS, can help to prevent the body from using endogenous sources of energy (lean body mass, adipose tissue), and it can help to restore positive energy balance during prolonged fasting, as described further below.

### 5.1. Energy

The primary function of dietary carbohydrate in ONS and other food products is to provide an energy source. Carbohydrate delivers roughly 4 kcal per gram, which is a slightly greater level of energy than delivered by protein and roughly half the calories per gram delivered by fat [47]. In a fed state, the heart, renal cortex, skeletal muscle, brain, and other neural tissues are preferential users of glucose for their energy needs. In contrast, red blood cells (RBCs), the most abundant cell type in the blood, rely explicitly on glucose for energy metabolism because they lack mitochondria (the cellular site for oxidative metabolism of fat). Without a consistent source of glucose, RBCs do not survive. RBCs are vital to the body, for they deliver oxygen from the lungs to body tissues—via the circulatory system—and then return carbon dioxide from the body to the lungs, where it can be exhaled [48]. In addition, cells of the renal medulla also rely explicitly on glucose for meeting their energy demands [49].

### 5.2. Preventing the Utlization of Endogenous Energy Sources during Prolonged Fasting

The post-absorptive state, or the time after eating when the body has absorbed the nutrients from the gut, occurs theoretically when the last nutrients of the fed state are used [49]. The limited reserves of glycogen (stored sugar) in the liver and skeletal muscle are depleted within the first 12 h of the post-absorptive phase [49]. The lack of available glucose in the post-absorptive phase triggers hormonal changes that signal the switch in metabolic pathways from glycolysis (the breakdown of glucose for energy) to glycogenolysis (the breakdown of glycogen), gluconeogenesis (the breakdown of non-carbohydrate substrates to form glucose), and lipolysis (the breakdown of fat) [49]. Thus, if carbohydrate is not available as a fuel source within this period, the metabolic pathways of starvation are initiated (as mentioned above), and the body is forced to use endogenous sources for energy.

Ultimately, prolonged fasting results in decreased protein synthesis and the increased catabolism of lean body mass (LBM) and adipose tissue. The breakdown of LBM will provide substrates for gluconeogenesis in the form of amino acids. Over a span of several days, LBM losses can equate to several kilograms of body weight loss—leading to the impairment of skeletal, respiratory, and cardiac muscle, compromised immunity, hepatic insufficiency, impaired GI tract motility, and maldigestion/malabsorption (due to decreased villi and crypts) [49]. Carbohydrate from food or ONS can help to spare protein, reduce the catabolism of LBM, and help to restore positive energy balance. Furthermore, the provision of carbohydrate can help prevent the mobilization of fat from adipose tissue, and it can prevent ketone body accumulation in patients at-risk for ketoacidosis [49].

## 6. Formulation Roles of Carbohydrate in ONS

Besides its functional role in the body, carbohydrate also plays an important role for the flavor and palatability of ONS products. In development of ONS formulations, one of the primary functions of carbohydrate is to provide sweetness to the product [19]. In addition to this capacity, carbohydrate imparts a wide range of food functionalities [45]. Depending on the type of carbohydrate, it can enhance positive flavor attributes, mask negative flavor attributes, and act as a chemical precursor to desirable flavor and color development that occurs during manufacturing. Some carbohydrates also impact the “mouthfeel” of a product by increasing viscosity and providing textural cues perceived by the somatosensory system. The numerous benefits of added sugars make them a vital formulation ingredient. It would be virtually impossible to eliminate or completely replace sugars from a food formulation without affecting the flavor quality of the product [7].

### 6.1. Flavor Enhancement and Masking

Aside from its direct contribution to sweetness, carbohydrate can also enhance or suppress flavor attributes, further affecting flavor perception and influencing palatability. Research on taste perception has revealed that sweetness imparted by sucrose and other sweeteners has been shown to enhance perception of fruit and chocolate flavors [50,51,52]. Sugars and other carbohydrates have also been shown to enhance the aroma of foodstuffs by affecting volatility [53,54,55] or by a phenomenon known as flavor congruency [56]. Congruency arises from cross-modal interactions between flavor stimuli across the multisensory systems. These cross-modal interactions lead to learned flavor associations and expectations. For example, odors and tastes normally found together can be perceived as one sensation, such as vanilla and sugar [57]. This process creates a taste perception association. In other words, when we smell vanilla, our brains will expect to perceive sweetness in conjunction with it.

In taste perception, sugars can also suppress the distinction of bitterness, sourness, and saltiness [58]. The nutrients found in ONS products, such as protein, vitamins, and minerals, can impart excessive bitterness and sourness, which are commonly considered negative flavor attributes, necessitating the use of sugars to mitigate the impact of the negative tastes and provide a more balanced flavor profile. Overall, the enhancing or masking functionality of sugars and other carbohydrates can help improve consumer preference [59], particularly as flavor is the most principal factor for compliance with ONS recommendations [19].

### 6.2. Color and Flavor Formation

During the common food manufacturing step of heating, carbohydrate promotes color and flavor formation primarily through two non-enzymatic browning mechanisms, caramelization and the Maillard reaction [60]. These non-enzymatic browning mechanisms occur during the manufacturing process of many ONS, resulting in favorable color, aroma, and taste changes that are typically preferred by individuals consuming ONS. Caramelization, or the oxidation of sugar, is a reaction that occurs during dry heating above temperatures of 110 °C [7]. This process will yield volatile compounds, which contribute to desirable aromas, and large polymers, which are responsible for brown colors and additional texture [61,62].

In addition to caramelization, sugars can impact flavor and color via the Maillard reaction [7]. The Maillard reaction was named after French chemist Louis-Camille Maillard, who, in 1912, originally observed and described the reaction between amino acids and reducing sugars [63]. It is generally recognized the Maillard reaction plays a predominant role in flavor development during the cooking of food. Simple sugars, or carbohydrate, are broken down during heating by reacting with amino groups, either from free amino acids or proteins, to initiate a cascade of complex reactions that ultimately leads to the generation of aroma compounds and melanoidins (brown pigments that impart color in foods). Melanoidins differ from caramel color due to the incorporation of amino groups in their structure. Flavor compounds generated include numerous classes of chemicals, which are produced by fragmentation of sugars, amino acids, or intermediate compounds and impart desirable sweet, brown, roasted, toasted, and nutty aromas [60]. The health effects of these components in the balance of an overall diet are beginning to be better understood. Today, more than 3500 volatiles have been identified as Maillard reaction products including aldehydes, ketones, nitrogen, sulfur containing heterocyclic compounds, and many more [64].

Neither of the important color or flavor development reactions could occur in the production of ONS without added sugars. Sugar substitutes have different chemical structures [65], different melting points [66], and different hygroscopic qualities [7] that do not necessarily lend themselves to these reactions, not to mention sugar substitutes often impart distinctive after-tastes [59]. Thus, another important function of sugar is its role as a flavor or color precursor, where it imparts several vital sensory attributes that consumers expect. Elimination of sugar or its replacement with a high-intensity sweetener would impede the development of the characteristic desired color and flavor of ONS and reduce the level of acceptance and, thus, compliance.

### 6.3. Texture

In liquid food products, such as ONS, sugar binds water and can increase viscosity, provide desired mouthfeel sensations or thickness, increase boiling temperature, decrease freezing temperature, decrease the water activity, and alter the behavior of proteins and starches [7]. All sugars, mono-, di-, oligo-, and polysaccharides, contain hydroxyl (–OH) groups that form hydrogen bonds with water and have the potential to alter the texture of foods by imparting viscosity, thickness and “mouthfeel” attributes [7]. In the absence of additional thickening agents, the viscosity of the liquid food is proportional to the number of hydroxyl groups in the mix, and therefore, it is proportional to the amount of carbohydrate in the product [7]. For texture, carbohydrate is a remarkable natural substance that contributes to a myriad of functions that would require multiple synthetic compounds to do the same job in food product formulations [7].

## 7. Summary

A health policy framework focused on reducing added sugars in the general population does not translate effectively for the majority of at-risk or malnourished patients who need calories in a blend of protein, fat, and carbohydrate from ONS products. Added carbohydrate, or sugar, has indispensable roles within ONS in terms of flavor, function, and formulation, which cannot be replicated by other nutrients or constituents. Sugar is an important ingredient that adds positive sensory attributes to ONS by imparting desirable taste, aroma, color, and texture, all of which help to drive patient compliance. Lastly, as formulated nutrition products, ONS rely on sugars to provide a needed source of energy, which in turn prevents endogenous sources of energy (LBM and adipose tissue) from being used. Healthcare professionals need to work with nutritionally at-risk patients and their families to educate them about the purpose of added sugars in ONS.

## References

[1] Stratton R.J., Elia M. (2007). A review of reviews: a new look at the evidence for oral nutritional supplements in clinical practice. Clin. Nutr. Suppl..

[2] Engfer M., Green C. Oral Nutritional Supplement to Tackle Malnutrition: Summary Booklet. Medical Nutrition International Industry. http://www.file:///C:/Users/bronsal/Downloads/MNIBookletFINAL2911%20(2).pdf.

[3] Oral Nutritional Supplements The British Association for Parenteral and Enteral Nutrition Website. http://www.bapen.org.uk/nutrition-support/nutrition-by-mouth/oral-nutritional-supplements.

[4] Absorption of Monosaccharides Vivo Pathophysiology Website. http://www.vivo.colostate.edu/hbooks/pathphys/digestion/smallgut/absorb_sugars.html.

[5] Tierney M., Gallagher A., Giotis E.S., Pentieva K. (2017). An online survey on consumer knowledge and understanding of added sugars. Nutrients.

[6] Know Your Limit for Added Sugars Center for Disease Control and Prevention Website. http://www.cdc.gov/nutrition/data-statistics/know-your-limit-for-added-sugars.html.

[7] Clemens R.A., Jones J.M., Lee S.Y., Mayhew E.J., Slavin J., Zivanovic S. (2016). Functionality of sugars in foods and health. Compr. Rev. Food Sci. Food Saf..

[8] Dennis B. FDA Says Consumers Should Have More Details about ‘Added Sugars’ in Foods. Washington Post. https://www.washingtonpost.com/news/to-your-health/wp/2015/07/24/should-consumers-have-details-about-added-sugar-in-foods-fda-says-yes/.

[9] US Department of Health and Human Services, US Department of Agriculture (2010). 2010 Dietary Guidelines for Americans.

[10] World Health Organization WHO Calls on Countries to Reduce Sugars Intake among Adults and Children. http://www.who.int/mediacentre/news/releases/2015/sugar-guideline/en/.

[11] US Department of Health and Human Services, US Department of Agriculture (2015). 2015–2020 Dietary Guidelines for Americans.

[12] Guideline: Sugar Intake for Adults and Children World Health Organization. https://public.eblib.com/choice/publicfullrecord.aspx?p=2033879.

[13] Changes to the Nutrition Facts Label U.S. Food and Drug Administration Website. https://www.fda.gov/Food/GuidanceRegulation/GuidanceDocumentsRegulatoryInformation/LabelingNutrition/ucm385663.htm.

[14] Terrie Y.C. Medical Food and Meal Replacement Nutritional Supplements. Pharmacy Times Website. http://www.pharmacytimes.com/publications/issue/2015/january2015/medical-food-and-meal-replacement-nutritional-supplements?p=2.

[15] Therapeutic Abbott Nutrition Website. https://abbottnutrition.com/therapeutic.

[16] Dietary Reference Intakes for Energy, Carbohydrate, Fiber, Fat, Fatty Acids, Cholesterol, Protein, and Amino Acids Institute of Medicine. http://www.nationalacademies.org/hmd/Reports/2002/Dietary-Reference-Intakes-for-Energy-Carbohydrate-Fiber-Fat-Fatty-Acids-Cholesterol-Protein-and-Amino-Acids.aspx.

[17] Jensen G. (2013). Oral nutritional supplementation. Am. J. Manag. Care.

[18] Wilson M., Purushothaman R., Morley J. (2002). Effect of liquid dietary supplements on energy intake. Am. J. Clin. Nutr..

[19] Ruxton C. (2014). Compliance with Oral Nutritional Supplements and the Role of Taste. CN Focus.

[20] Hubbard G.P., Elia M., Holdoway A., Stratton R.J. (2012). A systematic review of compliance to oral nutritional supplements. Clin. Nutr. Edinb. Scotl..

[21] Darmon P., Karsegard V.L., Nardo P., Dupertuis Y.M., Pichard C. (2008). Oral nutritional supplements and taste preferences: 545 days of clinical testing in malnourished in-patients. Clin. Nutr..

[22] Breslin P.A.S. (2013). An evolutionary perspective on food and human taste. Curr. Biol..

[23] How Does Our Sense of Taste Work? PubMed Health Website. https://www.ncbi.nlm.nih.gov/pubmedhealth/PMH0072592/.

[24] Hardy K., Brand-Miller J., Brown K.D., Thomas M.G., Copeland L. (2015). The importance of dietary carbohydrate in human evolution. Q. Rev. Biol..

[25] Tastes Differ: How Taste Preferences Develop The European Food Information Council (EUFIC). http://www.eufic.org/en/food-today/article/tastes-differ-how-taste-preferences-develop.

[26] Auvray M., Spence C. (2008). The multisensory perception of flavor. Conscious. Cogn..

[27] Beauchamp G.K., Cowart B.J. (1985). Congenital and experiential factors in the development of human flavor preferences. Appetite.

[28] Hoffman J., Ishii E., Macturk R. (1998). Age-related changes in the prevalence of smell/taste problems among the United States adult population. Ann. N. Y. Acad. Sci..

[29] Cooper R.M., Bilash I., Zubek J.P. (1959). The effect of age on taste sensitivity. J. Gerontol..

[30] Stevens J.C., Cain W.S., Demarque A., Ruthruff A.M. (1991). On the discrimination of missing ingredients: Aging and salt flavor. Appetite.

[31] Stevens J.C. (1996). Detection of tastes in mixture with other tastes: Issues of masking and aging. Chem. Sens..

[32] Schiffman S.S. (1993). Perception of taste and smell in elderly persons. Crit. Rev. Food Sci. Nutr..

[33] Schiffman S.S. (1997). Taste and smell losses in normal aging and disease. JAMA.

[34] Duffy V.B., Backstrand J.R., Ferris A.M. (1995). Olfactory dysfunction and related nutritional risk in free-living, elderly women. J. Am. Diet. Assoc..

[35] Arcavi L., Shahar A. (2003). Drug related taste disturbances: Emphasis on the elderly. Harefuah.

[36] Schiffman S.S., Gatlin C.A. (1993). Clinical physiology of taste and smell. Annu. Rev. Nutr..

[37] Schiffman S.S., Graham B.G., Suggs M.S., Sattely-Miller E.A. (1998). Effect of psychotropic drugs on taste responses in young and elderly persons. Ann. N. Y. Acad. Sci..

[38] Douglass R., Heckman G. (2010). Drug-related taste disturbance. Can. Fam. Phys..

[39] Seiberling K.A., Conley D.B. (2004). Aging and olfactory and taste function. Otolaryngol. Clin. N. Am..

[40] MacDonald D.E. (2006). Principles of geriatric dentistry and their application to the older adult with a physical disability. Clin. Geriatr. Med..

[41] Wrobel B.B., Leopold D.A. (2004). Clinical assessment of patients with smell and taste disorders. Otolaryngol. Clin. N. Am..

[42] Imoscopi A., Inelmen E.M., Sergi G., Miotto F., Manzato E. (2012). Taste loss in the elderly: Epidemiology, causes and consequences. Aging Clin. Exp. Res..

[43] Jensen S.B., Mouridsen H.T., Bergmann O.J., Reibel J., Brünner N., Nauntofte B. (2008). Oral mucosal lesions, microbial changes, and taste disturbances induced by adjuvant chemotherapy in breast cancer patients. Oral Surg. Oral Med. Oral Pathol. Oral Radiol. Endod..

[44] Schiffman S.S. (2000). Intensification of sensory properties of foods for the elderly. J. Nutr..

[45] Mathey M.F., Siebelink E., de Graaf C., Van Staveren W.A. (2001). Flavor enhancement of food improves dietary intake and nutritional status of elderly nursing home residents. J. Gerontol. A Biol. Sci. Med. Sci..

[46] Zimmerman M., Snow B. The Functions of Carbohydrates in the Body. In An Introduction to Nutrition. https://2012books.lardbucket.org/pdfs/an-introduction-to-nutrition.pdf.

[47] How Many Calories Are in one Gram of Fat, Carbohydrate, or Protein? United States Department of Agriculture’s National Agricultural Library Website. https://www.nal.usda.gov/fnic/how-many-calories-are-one-gram-fat-carbohydrate-or-protein.

[48] American Society of Hematology Blood Basics. http://www.hematology.org/Patients/Basics/.

[49] Nelms M. (2017). Starvation, Underweight, Malnutrition and Eating Disorders. Lecture Presentation Slides.

[50] Bonnans S., Noble A.C. (1993). Effect of sweetener type and of sweetener and acid levels on temporal perception of sweetness, sourness and fruitiness. Chem. Sens..

[51] Valdes R., Hinreiner E., Simone M. (1956). Effect of sucrose and organic acids on apparent flavor intensity. Food Technol..

[52] VonSydow E., Moskowitz H., Jacobs H., Meiselman H. (1974). Odor-taste interaction in fruit juices. Lebensm. Wiss. Technol..

[53] Chen S.-D., Ofoli R.Y., Scott E.P., Asmussen J. (1993). Volatile retention in microwave freeze-dried model foods. J. Food Sci..

[54] Sugisawa H., Kobayashi N., Sakagami A. (1973). The retention of volatile flavors in food. Jpn. Soc. Food Sci. Technol..

[55] Ehler K.F., Bernhard R.A., Nickerson T.A. (1979). Heats of adsorption of small molecules on various forms of lactose, sucrose, and glucose. J. Agric. Food Chem..

[56] Schifferstein H.N., Verlegh P.W. (1996). The role of congruency and pleasantness in odor-induced taste enhancement. Acta Psychol..

[57] Green B.G., Nachtigal D., Hammond S., Lim J. (2012). Enhancement of retronasal odors by taste. Chem. Sens..

[58] Keast R.S.J., Breslin P.A.S. (2003). An overview of binary taste–taste interactions. Food Qual. Prefer..

[59] Goldfein K.R., Slavin J.L. (2015). Why sugar is added to food: Food science 101. Compr. Rev. Food Sci. Food Saf..

[60] Kerler J., Winkel C., Davidek T., Blank I., Taylor A.J., Linforth R.S. (2010). Chapter 3: Basic chemistry and process conditions for reaction flavours with particular focus on maillard-type reactions. Food Flavour Technology.

[61] Monte W., Maga J. (1982). Flavor chemistry of sucrose. Sugar Technol. Rev..

[62] Colonna W., Samaraweera U., Clarke M., Cleary M., Godshall M., White J.S. (2006). Sugar. Kirk-Othmer Encyclopedia of Chemical Technology.

[63] Billaud C., Adrian J. (2003). Louis-camille maillard, 1878–1936. Food Rev. Int..

[64] Reineccius G. (2005). Flavor Chemistry and Technology.

[65] Chattopadhyay S., Raychaudhuri U., Chakraborty R. (2014). Artificial sweeteners—A review. J. Food Sci. Technol..

[66] Dhartiben B.K., Aparnathi K.D. (2017). Chemistry and use of artificial intense sweeteners. Int. J. Curr. Microbiol. App. Sci..

